# Control and consequences of IL-6 receptor ectodomain shedding

**DOI:** 10.1186/2047-783X-19-S1-S17

**Published:** 2014-06-19

**Authors:** Jürgen Scheller

**Affiliations:** 1 Institute of Biochemistry and Molecular Biology II, Heinrich Heine University, 40225 Düsseldorf, Germany

## Background

Interleukin-6 type cytokines are mainly involved in inflammation by controlling differentiation, proliferation, migration, and apoptosis of target cells. A dysfunction of the complex regulatory cytokine network might lead to acute and chronic inflammation, autoimmune diseases or neoplastic disorders. IL-6 deficient mice were found to be resistant to collagen- and antigen-induced arthritis, highlighting the role of IL-6 in chronic inflammation and autoimmune diseases. The IL-6 receptor complex consists of the signal-transducing gp130 receptor and the non-signaling IL-6 receptor (IL-6R) which exists in membrane bound and soluble forms. A soluble form of the human IL-6R (sIL-6R) is mainly generated by limited proteolysis (ectodomain shedding) but also by alternative splicing. IL-6 signaling via the membrane-bound IL-6R and gp130 is called classic signaling, whereas IL-6 signaling via the soluble IL-6R and gp130 is referred to as IL-6 trans-signaling. Gp130 is ubiquitously expressed, whereas the membrane-bound IL-6R is mainly expressed on lymphocytes and hepatocytes. Therefore, IL-6 trans-signaling virtually expands IL-6 signaling to all cells of the body. Using the specific trans-signaling inhibitor soluble gp130 (sgp130), we showed that IL-6R ectodomain shedding which facilitates IL-6 trans-signaling is the crucial step in the development and the progression of chronic inflammatory disorders and inflammation-induced cancer.

## Structural requirements of IL-6R ectodomain shedding

Most post-transcriptional modifications such as phosphorylation, which control the fate and activity of proteins, are completely reversible. Proteolysis is exceptional because of its irreversible nature. The proteolytic release of membrane-anchored proteins was also called (ectodomain) shedding, and the responsible proteases were termed “sheddases” [1].

Ectodomain shedding of the IL-6R has two functional consequences. First, loss of membrane-bound IL-6R renders the cell non-responsive towards IL-6 classic signaling, thereby abrogating IL-6 signaling. Paradoxically, the generated soluble IL-6R (sIL-6R) itself is biologically active and can mediate IL-6 trans-signaling on cells expressing gp130. A high concentration of 25–50 ng/ml sIL-6R is found in the serum of healthy humans. Under pathophysiological conditions, sIL-6R levels rise up to three fold. The cellular source of the sIL-6R and the *in vivo* mechanism of its generation are still unclear. The vast majority (90–99%) of the sIL-6R should originate from ectodomain shedding of the membrane-bound precursor, whereas alternative splicing of the IL-6R mRNA is accounts only for a minor proportion (1–10%) [1].

The sIL-6R is generated by constitutive and induced shedding by the “A disintegrin and metalloproteinases” (ADAM)10 and ADAM17 [1]. Recently, we characterized the structural requirements of IL-6R shedding on the site of the substrate [2]. The IL-6R consists of three extracellular domains, important for efficient exocytosis and IL-6 binding. The extracellular domains are followed by a flexible, 52 amino acids long stalk region, a trans-membrane domain and an intracellular domain which regulate basolateral sorting of the IL-6R [3]. The ADAM17 cleavage site was identified within the stalk region between the amino acids 357Q and 358D [1]. Deletion of 10 amino acids surrounding the ADAM17 cleavage site abrogated ADAM17 shedding of the IL-6R but leaves ADAM10 shedding intact. This suggested that the ADAM10 cleavage site is located at a different site. Interestingly, ADAM proteases do not have defined cleavage consensus sequences, which hinder the identification of novel cleavage sites by substrate sequence analysis. Deletion of 5 additional juxtamembrane located amino acids in the aforementioned delta10 IL-6R variant abolishes also ADAM10-mediated ectodomain shedding of the IL-6R [2], suggesting that the ADAM10 cleavage site is in close proximity to the ADAM17 cleavage site. Importantly, both IL-6R variants (delta10 and delta15) are biologically active. Moreover, we showed that only about 20 of the 52 amino acids of the IL-6R stalk region are needed for biological activity of the IL-6R, which supported a model of the IL-6/IL-6R/gp130 signal transducing complex, in which the gp130 receptor chain has a C-shaped structure after IL-6/IL-6R/gp130 complex formation [2]. In this model, the stalk region is necessary for the correct positioning of the IL-6-binding domains of the IL-6R in the signal transducing receptor complex.

## Constitutive shedding is dependent on the level of cellular IL-6R expression

Apart from induced IL-6R shedding, which is activated by various substances and conditions, including phorbol esters such as phorbol-12-myristat-13-acetate (PMA), the Ca^2+^ ionophor ionomycin, extracellular ATP, low membrane-cholesterol levels and apoptosis [1], the IL-6R is also constitutively released from the cell surface, without obvious cellular stimulation. Here, we demonstrated that cellular senescence and EGF-R stimulation lead to increased IL-6R expression, which was regulated via the mTOR pathway [4]. The simple increase in cell surface expressed IL-6R also led to increased generation of sIL-6R, suggesting that the expression level of the cellular substrate indirectly determines the amount of constitutive shedding. sIL-6R serum levels are increased under various inflammatory conditions and up to now, it is not clear if this is dependent on increased IL-6R expression, induced or constitutive shedding. Or data, however, open up the possibility that both mechanisms contribute to inflammation-induced increased sIL-6R levels.

## Conclusions

In recent years, we characterized the biological functions of IL-6 trans-signaling and sgp130, which specifically suppresses overshooting IL-6 trans-signaling activities but to leaves “beneficial” IL-6 classic signaling intact. The biological sgp130Fc is currently tested in phase I clinical trials [5]. In contrast to treatment with neutralizing TNFalpha antibodies, administration of the trans-signaling inhibitor sgp130 did not interfere with protective immune responses after infection with *Mycobacterium tuberculosis* in mice [3]. Moreover, classical signaling was sufficient for early control of *Listeria monocytogenes* infection in mice [6]. Blockade of IL-6 trans-signaling might therefore exhibit advantages as compared to the global blockade of IL-6 or TNFalpha by monoclonal antibodies for the treatment of chronic inflammatory diseases.
